# Infection prevention knowledge and perceptions: a nationwide survey among nurses and physicians in adult intensive care units in Finland

**DOI:** 10.1371/journal.pone.0325323

**Published:** 2025-06-18

**Authors:** Kirsi Terho, Eliisa Löyttyniemi, Esa Rintala, Sanna Salanterä

**Affiliations:** 1 UniTurku, Department of Nursing Science, Department of Hospital Hygiene and Infection control, Turku University Hospital, University of Turku, Turku, Finland; 2 Department of Hospital Hygiene and Infection control, Turku University Hospital, Turku, Finland; 3 Department of Biostatistics, University of Turku and Turku University Hospital, Turku, Finland; 4 UniTurku, Department of Nursing Science, Turku University Hospital, Administration, Nursing, University of Turku, Turku, Finland; CHUV: Centre Hospitalier Universitaire Vaudois, SWITZERLAND

## Abstract

**Background:**

Healthcare-associated infections are a major complication of care for patients in intensive care, causing costs and additional mortality. Infection prevention practices, such as hand hygiene, have been suboptimal globally. This study aimed to explore the level of knowledge and perceptions of critical care staff regarding healthcare-associated infections as insufficient knowledge contributes to an increased burden of these infections.

**Methods:**

A nationwide survey of physicians and nurses working in intensive care units of Finnish tertiary care hospitals was conducted to gain knowledge and explore perceptions regarding the prevention of healthcare-associated infections in intensive care units. Descriptive statistics were used to describe the study data, and a mainly nonparametric method was used to compare the groups.

**Results:**

The respondents demonstrated moderately good knowledge of hand hygiene and infection prevention, with a median of 36 correct responses (Q1, Q3: 34, 37). However, there were notable gaps in their knowledge in infection prevention regarding the routes of infection transmission, with a median score of 4 (Q1, Q3: 4, 6). Conversely, perceptions of infection prevention were generally positive. The median score for perceptions was 51 (Q1, Q3: 47, 55), but no significant association was found between perceptions and knowledge levels.

**Conclusions:**

The level of knowledge about healthcare-associated infections is not satisfactory. In particular, there is a lack of in-depth understanding of the mechanisms of infection transmission and prevention. Providing unit-tailored feedback on performance, along with education on the transmission mechanisms and infection prevention for healthcare workers is essential.

## Introduction

Healthcare-associated infections (HCAIs) remain a major factor that impairs patient outcomes, causing mortality, suffering, and costs, especially in intensive care [1]. Infection prevention and control (IPC) involves various elements. Of those elements, hand hygiene (HH) remains the single most effective measure in preventing HCAI, and improving HH has been shown to reduce HCAIs. Compliance with IPC practices, especially HH, is suboptimal [2, 3, 4].

Previous research shows remarkable variation in IPC and HH practices among institutions and between individuals [5]. According to one review of HH in intensive care units (ICUs), compliance ranged from 9.1%–64.5%, depending on the country, specialty, and profession of the staff [6]. Similar results were found in a more recent study on individual practices across Europe [5]. Compliance can be influenced by the clinical environment, such as the availability of hand sanitizers and guidelines for HH techniques [7, 8, 9]. Organizational and resource factors, such as the nurse-patient ratio can also affect HH compliance: higher ratios are associated with higher HH compliance [5]. Communication factors can also play a role in HH practices such as professional information sharing and feedback [7].

Implementing IPC and HH practices are individual decisions based on knowledge and attitudes [10]. Greater knowledge of IPC and understanding HH can influence attitudes and increase compliance among hospital staff [4,11]. Knowledge gaps regarding HH have been identified among healthcare personnel [7,11,12], although satisfactory level of knowledge has also been reported [4]. What is clear, however, is that the ability of nurses to identify IPC problems and take responsibility has a positive effect on compliance [13]. Even though nurses have been observed to have positive attitudes towards HH, suboptimal practices have also been identified [7,14]. Compliance with HH has been seen time-consuming by nurses, and they have seen the aim of HH as being self-protection [14,15]. In some cases, the personal compliance of HH has been overestimated [16,17].

Organizational culture and leadership also significantly impact IPC and HH performance [8,18]. Research has shown that a positive leadership attitudes, commitment, open discussions, and a supportive atmosphere improve HH compliance [8,19,20]. Further, HH practices tend to be more compliant with regulations when managers have expressed that non-compliance is not tolerated. Management-initiated IPC development activities and providing feedback have also shown to improve HH compliance [21, 22, 23]. In addition, the social context of the work environment, including acceptance of IPC behaviors, role models, and peer or workplace commitment to IPC, appear to influence compliance [7,18,22,24].

Various interventions, including training, have been implemented to improve IPC and HH practices [25,26]. Unfortunately, optimal levels of adherence to IPC practices have not been achieved, and the impact has often been short lived, despite repeated education [23,27]. In 2009, the World Health Organization (WHO) launched a multimodal strategy and campaign [28]. In line with this strategy, several IPC programs, including HH improvement projects, have included interventions such as performance monitoring, feedback, workplace reminders, education, and training [29, 30, 31, 32]. Despite extensive research, further studies are needed to appropriately target prevention interventions [1]. Additionally, although the knowledge and perceptions of infection control in intensive care units have been studied in several countries, nationwide studies are scarce.

Therefore, this study investigates the knowledge and perceptions of IPC, including HH, among nurses and physicians in various intensive care units (ICUs) at the national level in Finland. It also examines the impact that the number of beds and the number of nurses and physicians working in units can have on HH knowledge and perceptions.

## Methods

### Design

We conducted a cross-sectional nationwide survey in Finnish intensive care units (with the exception of the island of Åland). We surveyed a total of 25 ICUs. The number of personnel at that time was 1625.

We invited all registered nurses and physicians in all Finnish (non-Ålandic) ICUs to participate. We wanted to give all personnel the possibility to answer a self-administered survey; therefore, no sampling procedure was in place. The response period was three to four weeks in February and March 2017. One of the ICUs we contacted did not send back responses. All ICUs in Finland are publicly owned.

The survey is reported according to the Checklist for Reporting of Survey Studies (CROSS) [33].

### Participants

All nurses and physicians whose main place of work was in an adult intensive care unit were included. Employees who only consulted in the ICU were not involved in the survey. The survey included healthcare personnel in managerial positions, which could be a physician or nurse who had administrative tasks in addition to their professional work. Information on the total number of staff was gathered from the head nurse of each ICU.

### Data collection tool

The questionnaire consisted of WHO’s perception questionnaire (Perception Survey for Healthcare Workers [revised August 2009]) and knowledge questionnaire (HH Knowledge Questionnaire for Healthcare Workers [revised August 2009]) [34]. The original instruments were translated (and back translated) by a professional language translator (Lingsoft Language Services Oy). The translated instruments were tested with an IPC professional and two nurses in intensive care. After testing, minor linguistic corrections related to the Finnish way of presenting the issue were made.

The questionnaire included socio-demographic questions (eight–nine questions), questions on IPC and HH knowledge (22 items), and questions on perceptions towards IPC and HH (22 items).

With three open questions, participants were asked to assess the proportion of maintained HH and the proportion of healthcare-associated infections.

For the knowledge questions, the response options were generally yes/no (12 items), or the correct answer could be selected from the options provided (10 items). Participants were asked to express their opinions or assess the items presented. Perceptions were measured using semantic differential scales constructed from bipolar adjectives that ranged from no impact or importance to very effective or very high importance. These items were measured with either a seven-level scale (17 items) or a four-level scale (5 items).

### Survey administration

After receiving the research permits, we contacted the heads of the units, nurses, and physicians in each ICU directly by email and telephone. With their help, a contact person was appointed for each unit from 9 to 26 January 2017 to provide information to the participants and make practical arrangements for the study. From 6 to 9 February 2017, questionnaires in paper form were sent in a cardboard box to the contact person, who placed them in an agreed-upon designated place (often a box in the breakout room of the ICU). The box was sealed so that the questionnaires could remain anonymous, and the responses were collected from the ICU. The researcher’s address was written on the box so that the contact persons could return the box with the questionnaires by post directly to the researcher.

The questionnaire was accompanied by a form for the participants describing the study and their opportunity to voluntarily to participate. Nurses and physicians were asked to complete the questionnaire at their convenience. Participation in the survey was voluntary and anonymous. Contact persons were contacted by email and telephone during the study to check progress and provide support.

The anonymity of the participants was protected by the fact that the questionnaires were returned to a sealed box, which the unit contact person sent directly to the researcher by post after the completion of the data collection. The response data were saved in an Excel file by a person outside the study, and the documents were kept in a locked box for possible data checks.

### Data management

The WHO knowledge and perception questionnaire treated the responses as knowledge or perceptions based on the WHO model [34]. Participants responded to the knowledge questions using a scale of 0–1, with 1 being the correct answer. We organized the responses to the knowledge questions into groups: all questions, questions regarding to whom HH prevented the transmission of microbes and questions about the specific HH required for each procedure.

The four-point perception questions on the impact or importance of HH were first converted to a binary scale of perceived importance or insignificance. The four-point perception questions were converted into a seven-value scale (1- > 0, 2- > 2, 3- > 4, 4- > 6). This allowed all perception questions to be treated on the same scale, to be comparable, and to calculate the sum score for comparison. Naturally, it was not possible to calculate a sum score if any of the answers were missing. This had no practical effect on our conclusions since the number of missing responses was very low.

The perception questions were grouped into categories such as the perceived impact of the social environment or the importance of HH for the patient or organization. The answers to these questions were categorized into two categories (0–4 described non-important, and 5–6 described important). In addition, perception and knowledge answers were categorized according to correct answers (less than 80% answers correct, ≥ 80% correct). Participants who answered less than 80% of the knowledge questions correctly were classified as the “poor” group, while those who answered 80% or more correctly were classified as the “good” group. For the perception questions, participants who rated less than 80% of the items as important or effective for infection prevention were categorized as having “non-supportive perceptions of IPC”, while those who rated 80% or more were classified as having “supportive perceptions of IPC”.

We looked at the association between the size of each ICU and the proportion of correct answers to the knowledge questions (grouped by ≥ 80% correct answers, < 80% correct answers). ICU sizes were grouped by the number of nurses, the number of physicians, and the number of nurses divided by the number of patient beds. Table 1 gives further details on how we grouped participants.

**Table 1:** Background information of participants.

	Nurses n = 704 (88.2%)	Physicians n = 94 (11.8%)	Missing values (n)	Total 810
In managerial position	5.3% (37/704)	32% (30/94)	12	8% (67/798)
Age years: Md, (Q1, Q3), range years	40, (31,50), 23–64	41, (35,55), 27–65	12	40, (32,50), 23–65
Working time in the current position, years: Md, (Q1, Q3), range	12, (5,20), 0–39	6, (3,15), 0–32	8	12, (5,20), 0–39
Working time in the current unit, years: Md, (Q1, Q3), range	9, (3,16), 0–39	6, (1,15), 0–36	9	8, (3,16), 0–39
Formal training on HH in the last three years: proportion, CI 95%	66% (467/698), 63–70%	50% (47/94), 40–60%	8	65% (520/ 802), 61–68%
Used hand rub routinely in their work: proportion, CI 95%	99% (697/703) 98–99.6%	100% (94/94), 96–100%	3	99% (801/807), 98–100%

We also categorized working years in the current ICU into six groups: 1 (less than 1 year), 2 (1–3 years), 3 (3–4 years), 4 (5–9 years), and 5 (10–19 years), 6 (20 years or more). We categorized age into six groups: 1 (20–29 years old), 2 (30–39 years old), 3 (40–49 years old), 4 (50–59 years old), and 5 (60 years old or older).

### Data analysis

Categorical variables were summarized with counts and percentages. In addition, seven-step categorical variables—ineffective versus highly effective semantic differential scales—were handled as continuous variables (from zero to six), summarized with the mean with standard deviation (SD). Differences in responses between groups (e.g., physicians versus nurses, age groups, managerial position (no/yes) were examined using the Wilcoxon rank sum test and the Kruskal-Wallis test (when more than two groups were compared). Fisher’s exact test was used to study the association between responses of two dichotomic variables.

The Cochran-Armitage test for trend association was used between a variable with two categories and an ordinal variable with two or multiple categories. Spearman correlation coefficients test was used to test the correlation between variables. P-values (two-tailed) less than 0.05 were considered statistically significant. Statistical computations were carried out using the JMP^®^, Pro 17.0.0 for Mac. SAS Institute Inc., Cary, NC, 1989–2023.

### Ethical considerations

Permission to conduct the study in the ICUs was sought separately from the management of each critical care hospital according to each hospital’s approval policy. The Human Subjects Ethics Committee of the University (21/2016) approved the study’s ethics.

Participation in the survey was voluntary. There were no controls on the collection or return of the questionnaire. The work units did not know which personnel had participated and which had not. The identities and places of work of the participants remained anonymous throughout the study.

## Results

### Study participants

A total of 24 ICUs participated, and there were 3–26 hospital beds in the various ICUs. The total number of staff in the participating ICUs ranged from 19 to 180, and the number of working physicians ranged from 1 to 24. The survey was returned by 810 participants. The participants represented approximately 50% (810/1,625) of all ICU physicians and nurses in Finland. All responses had some missing values, including age, gender, and profession. The majority of participants were females (85%, 685/798). A detailed description of the study participants is given in Table 1.

### Knowledge of infection prevention and hand hygiene

The median knowledge score for participants was 36 (score range 0–44, Q1, Q3; 34, 37), with 7% (60/810) having at least one missing response. Overall, 66% (454/698) of participants demonstrated good knowledge. There was a significant difference in the mean level of knowledge between different ICUs (p = 0.004, Kruskal-Wallis test) with means from 33.8 to 36.2. We also found, that as the size of the ICU increased (number of physicians or beds), the number of correct answers also increased (Table 2).

**Table 2:** Proportion of correct answers in ICUs.

	Size of ICU	Number of ICUs, number of participants	Number of values	Good knowledge % (n)	Poor knowledge % (n)	p^*^
Number of nurses	10–30	4, 88	70	61% (43)	39% (27)	0.038
	31–50	10, 258	229	65% (150)	34% (79)	
	51–90	7, 201	164	59% (97)	41% (164)	
	90 <	4, 263	226	73% (164)	27% (27)	
Number of physicians	1	4, 102	93	66% (61)	34% (32)	0.142
	2–5	14, 384	321	63% (203)	37% (118)	
	> 6	7, 324	275	69% (190)	31% (85)	
Number of hospital beds	1–5	6, 140	124	58% (72)	42% (52)	0.0081
	6–10	11, 335	286	65% (186)	35% (100)	
	11 <	7, 335	279	70% (196)	30% (83)	
Number of nurses concerning beds	< 4	2, 60	57	62% (37)	35% (20)	0.10
	4–5	8, 207	177	64% (113)	30% (64)	
	5 ≤ 6	4, 86	66	62% (41)	25% (38)	
	6 ≤ 7	5, 159	135	65% (85)	37% (50)	
	7 ≤ 8	3, 156	133	71% (95)	38% (29)	
	8 <	3, 142	121	66% (83)	34% (38)	

* Cochran-Armitage Trend Test. Good knowledge = Respondents with at least 80% correct answers; Poor knowledge = Respondents with less than 80% correct answers.

**Fig 1:**
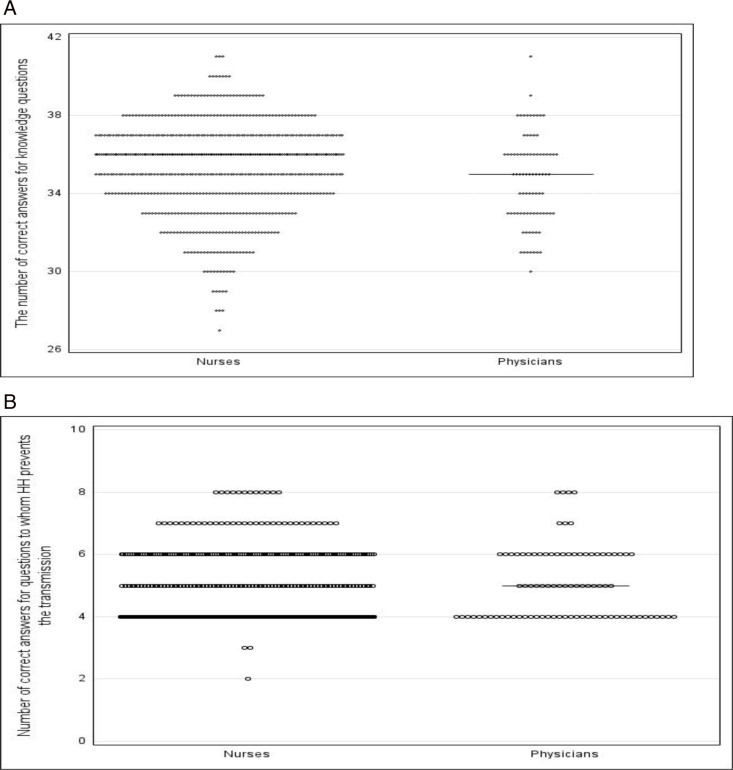
A. The number of correct answers in all knowledge questions (score range 0–44). **B. Number of correct answers on HH to prevent microbe transmission (the score range 0–8)**.

We assessed the relation between background information and the level of knowledge. Not even HH training received in the previous three years was found to have had an influence. Responding nurses had statistically higher knowledge scores on average than physicians (Fig 1 A).

We more closely assessed the questions concerning knowledge of HH and which HH actions prevent the transmission of germs to patients or healthcare workers (Table 3).

**Table 3:** Proportion and medians of correct answers in knowledge patterns.

Topic (number of questions)	The maximum score	Proportion of participants with good knowledge	Missing values	Nurses median; Q1, Q3	Physicians median; Q1, Q3	^*^ p
All knowledge questions of HH and IPC [44]	44	66% (454/689)	121	36; (34, 37)	35; (33, 36)	0.013
Knowledge of the factors hindering the performance of the HH [4].	4	94% (743/792)	18	4; (4, 4)	4; (4, 4)	0.011
Required HH method in different care situations [18].	18	91% (728/797)	13	17; (15, 17)	16; (15,17)	0.130
HH, which prevents the transmission of microbes to staff or patients.	8	7% (52/750)	60	4; (4, 6)	5; (4, 6)	0.0028

* Wilcoxon rank sum test.

Good knowledge = Respondents with at least 80% correct answers; Poor knowledge = Respondents with less than 80% correct answers.

The mean score for participants was 4.7 (score range 0–8, CI 95% 4.67 to 4.82). Only 2% (16/750) answered all questions correctly. The sum of the correct answers was slightly associated with gender (median score female 4 vs. males 5, p = 0.0028, Wilcoxon rank sum test) and profession. Additionally, the sum of correct answers correlated negatively with age (r_s_ = −0.12 p = 0.0012, Spearman correlation) and years of work in the ICU (r_s_ = −0.09 p = 0.0086, Spearman correlation) (Fig 1B).

An assessment of the number of HCAIs was provided by 63% (513/810) of participants. The mean of the estimated HCAI rate was 27% (SD 16.0; range 0–80). It was also noticed that 37% (302/810) of participants could not give an estimation as they selected the option “I do not know.” Of these, 27% (18/67) held managerial positions. Almost all the participants (99.9%; 805/806) evaluated HH as effective in preventing healthcare-associated infections.

### Perceptions of infection prevention and hand hygiene

Participants had supportive perceptions for IPC and HH, with a median score of 51 (Q1, Q3; 47,55), range 25–65), (score range 0–66, scale 0–6 for individual items). The perception of the importance of IPC and HH varied between the hospitals, with mean scores ranging from 10.3 to 11.6 out of 12. This difference was statistically significant (p = 0.012, Kruskal-Wallis test). There was no statistical significance in perceptions when looking at the size of the ICU (number of staff or patient beds in the ICUs).

Positive perceptions were expressed by 43% (324/759) of the participants. Among women, 45% (291/643) scored above 80% of the maximum score, compared to 29.0% (33/114) of men, the difference being statistically significant at p = 0.008 (Fisher’s exact test). Among those who had received HH training in the previous three years, 70% (223/320) had a more positive perception of IPC and HH (p = 0.014, Fisher’s exact test) than those who had not received training. There were also differences by age group, with less than 24% (31/128) of those under 30 and 53% (189/355) of those aged 40–60 scoring above 80%. This difference was also statistically significant (p < 0.001, Cochran-Armitage trend test). A higher percentage of the nurses (44%, 294/662) perceived the importance more strongly than the physicians (30%, 26/88). This difference was statistically significant (p = 0.008, Fisher`s exact test).

### Perceptions on the role of hand hygiene

Almost all participants felt that HH was important in their organization, both at the unit level (95%; 768/805) and the hospital level (92%; 740/806). The impact of HCAIs and HH was considered an important factor that influenced patient outcomes (98%; 794/806) and hospital costs (98%; 791/806). There was no significant difference between the perceptions of nurses and physicians (patient outcome p = 0.19, Fisher’s exact test; hospital costs p = 0.40, Fisher’s exact test). Women (93%) were slightly more likely than men (87%) to rate compliance with HH as important to the hospital (p = 0.045, Fisher’s exact test). Neither age nor years working in the ICU affected this perception. Working in a managerial position was also not related to perceptions of the importance of HH.

**Fig 2:**
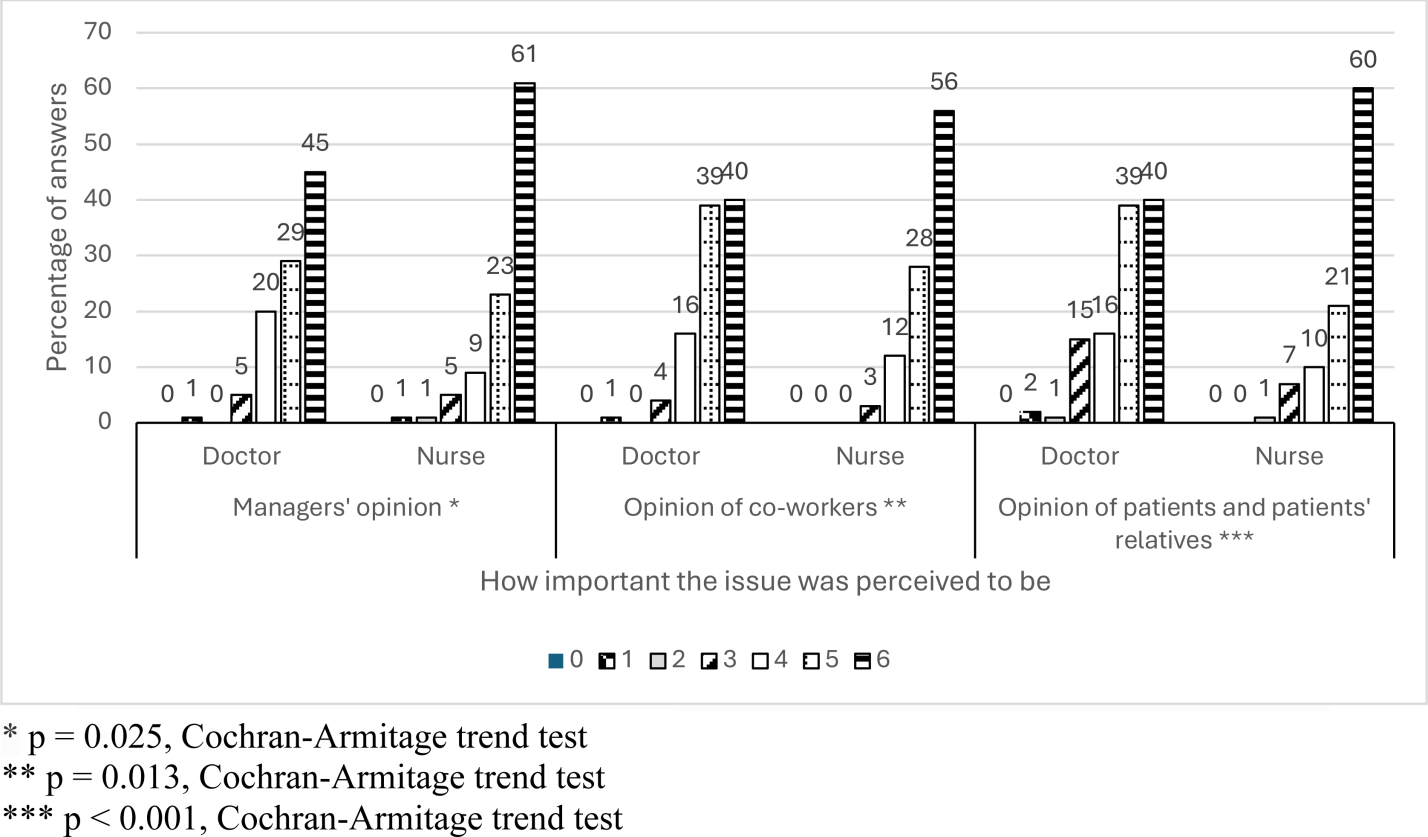
Nurses and physicians rated the performance of HH by superiors, colleagues, and patients/ relatives.

The opinions of others, such as supervisors, colleagues, and patients, on their own HH, were perceived as important. Nurses perceived the opinion of others as more important than physician did (p = 0.009, Wilcoxon rank sum test). In comparing the responses between participants of different age groups and with different amounts of working years (both as categorized), older age groups or those with more working experience tended to place a greater importance on the opinions of others in regard to HH (Kruskal-Wallis test: *p* < 0.001 for age groups, *p* = 0.011 for working experience) (Fig 2).

**Fig 3:**
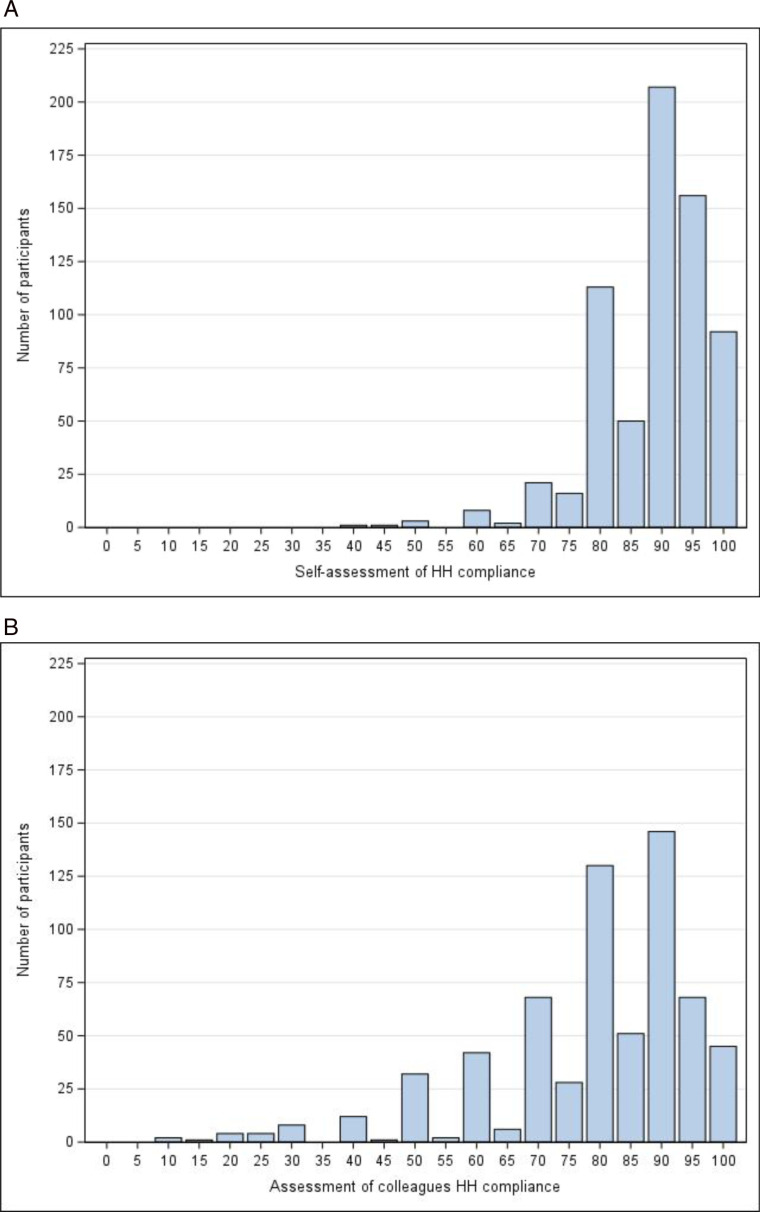
A. Proportion of situations requiring HH with self-assessed HH performance (0–100%). **B. Participants opinions on the HH compliance of an average collegue working in their unit (0–100%)**.

Participants rated self-perceived HH compliance as high, with a median of 90% (Q1 85%, Q3 97%; range 40–100%), but they rated others’ compliance as lower than their own (median 85%; Q1 70%, Q3 90%; range 0–100%) (Fig 3 A and Fig 3B).

A statistically significant positive correlation was found between the estimate of participants’ own HH compliance and their estimate of the HH performed by others (r_s_ = 0.63, p < 0.001 Spearman correlation). The participants’ perceptions of their own HH compliance were, on average, 10% (CI 95% 10.0 to 11.0) higher than their perceptions of the HH compliance of others (p < 0.0001, Wilcoxon signed-rank test). In addition, 40% indicated no or very little need for additional efforts in HH implementation, and only 8% thought others required additional effort. However, only 39% (313/795) of participants felt that their colleagues set a good example of HH to others. Participants evaluated various HH improvement interventions and HH education (76.4%; 617/808) as effective. The practices of more experienced colleagues in the organization, serving as role models for proper HH practices, were viewed favorably (72.4% 583/805) and how they perceived their own HH performance as being a good example were considered effective 69.8%, 559/801). They evaluated patient feedback on HH compliance and posters displaying information about HH as the least effective measures for improving HH in the hospital.

### Association between knowledge and perceptions

When the sum of the knowledge responses was compared to the sum of the perception responses, there was no significant correlation between them. In contrast, when looking at the questions asking respondents to assess who was primarily protected by HH, there was a negative correlation between the knowledge-level and perception-level sum variables (r_s _= − 0.13, p = 0.0004, Spearman correlation). The correlation between IPC and HH perceptions and the estimates of one’s own or others’ HH compliance was not strong. However, both were statistically significant (own r_s_ = 0.13, p = 0.0005 and others r_s_ = 0.16, p < 0.0001, Spearman correlation).

## Discussion

In our national study, the respondents’ knowledge of ICP and HH was insufficient, when only very high level of knowledge would be acceptable. Even so, perceptions towards IPC were quite positive. Overall, we found no correlation between IPC and HH knowledge and perceptions. However, we found a weak correlation between perceptions and knowledge about HH and the target of prevention at different moments of care.

According to previous studies, one barrier to infection prevention is a lack of knowledge [35]. However, in our study, HH training had no impact on the level of knowledge. We set the threshold for good knowledge at getting over 80% of the answers correct, and 66% of respondents reached this level. This result reflects knowledge gaps found in previous studies. For example, in a study using the same WHO questionnaire, nurses had an average of 66% correct answers [36], and in another study 66% of participants showed good knowledge of HH [12]. A qualitative interview study identified gaps in HH knowledge [7], and in a survey of ICUs in Europe (Italy), only 53% of participants correctly answered questions related to the prevention of infectious diseases [11]. On the other hand, a recent systematic review concluded that HH knowledge is adequate [29].

In our study, the most correctly answered questions were whether hand rubbing with alcohol, hand washing, or no HH is required in different caring situations. In contrast, fewer correct answers were given for questions where the transmission spreading had to be assessed in different HH situations, supporting the findings of a previous study [36]. Presumably, healthcare workers do not understand who is protected by a certain HH actions. If this is a case, they also have difficulty applying the correct HH actions to different care situations, and instead implementation is based only on learned phrases. Physicians had somewhat more correct answers to these questions than nurses, which may be related to physicians having more education about transmission mechanisms, although nurses may have received more HH training than other professional groups [37,38].

According to one European-level study of HH compliance, there was a relationship between the nurse-patient ratio and HH in ICUs [5]. We were not able to perform such an assessment, but we did examine the impact of the size of the ICU, concerning the number of staff and number of beds, across various variables. An increase in the ICU’s size generally correlated with a higher number of correct answers related to HH and IPC. This phenomenon requires further research on training in different ICUs and the structures that support infection control practices, for example.

Our study found that perceptions of IPC and HH and their impact on patient safety, outcomes, and costs were very positive, particularly among caregivers, older age groups, and those who have received HH training in recent years. This positive view of the issue was already noted in an older study [11]. Hazni et al. [39] used the WHO questionnaire and found a strong correlation between IPC and HH perceptions and the HH compliance assessment.

Previous studies have found that the social environment, including positive role models and managerial support, has a particular impact on the compliance of nurses with infection control measures [8,17,40–45]. However, in our study, only 39% rated more experienced colleagues as role models of good HH practice. Globally, organizational support and leadership have been found to be very important for the quality of IPC and HH globally [18,46]. Research has found that, when a group leader practiced good HH, the HH compliance in the healthcare facility was 71%, but without the leader’s HH example, it was 29% [42,45]. In our study, a managerial position did not mean better knowledge of IPC and HH, which is in line with one survey conducted in Vietnam [47]. Unfortunately, in our study, a significant proportion of those in managerial positions replied that they could not estimate the number of HCAIs. A third of all participants said that they did not know how to estimate the incidence of HCAI, and several did not answer this/ these questions. In addition, the estimated HCAI rates had a relatively wide range. Perhaps feedback on infection surveillance was not being used as a measure of quality of care, and thus it did not encourage staff to work independently on quality in IPC, which previous research has shown to be important [13,18,48].

Participants’ self-assessment of HH compliance was high in our study. They rated their compliance higher than that of their colleagues (90.0% and 80.1% respectively). Similar findings have been reported in previous studies, but in a study by Kelsicikova et al. [16], participants rated their compliance as only 74% and that of their colleagues as 51%. Up to 13% reported that they could not assess their HH compliance or that of their colleagues. It would be helpful if healthcare workers have a realistic picture of their compliance. It can be assumed that the closer one’s assessment is to their actual compliance level, the better their compliance can be improved. If nurses and physicians rate their HH as good, they do not feel that they need to change it.

### Limitations

Our study had several limitations. First, those who participated in the surveys may have had a greater interest or knowledge in the topic than non-respondents would have. Although the study used WHO questionnaires that had been translated and tested by professionals, participants may have interpreted some questions differently or there may not have been suitable response options available. In addition, the questionnaire was quite long, and some participants may not have fully engaged with all the questions due to the lack of interest or time. Some questions, such as those on infection rates or HH compliance, were perceived as difficult, leading to non-responses. We also did not have the ability to ensure that each respondent only answered the survey once. The ICUs were categorized based on the researchers’ judgement, and structural differences between ICUs were not addressed. Finally, cultural and structural differences between healthcare units may limit the generalizability of the results.

## Conclusions

There are gaps in the level of knowledge of IPC and HH between nurses and physicians in ICUs in Finland. In particular, the perception of one’s own compliance with HH is unrealistically high. There is a lack of deeper understanding on how to prevent infections through HH. HCAI surveillance data are poorly used to prevent HCAI and to motivate staff. Future studies should look at HCAI surveillance data and infection pathways and compare them with the level of knowledge and perceptions of professionals.

## Supporting information

S1 The infection prevention and hand hygiene questionnaire. (TIF)
