# Supplementary figures and images for: Infection prevention knowledge and perceptions: a nationwide survey among nurses and physicians in adult intensive care units in Finland
Source: PLoS One. 2025 Jun 18;20(6):e0325323. doi: 10.1371/journal.pone.0325323 (PMC12176175; doi:10.1371/journal.pone.0325323)

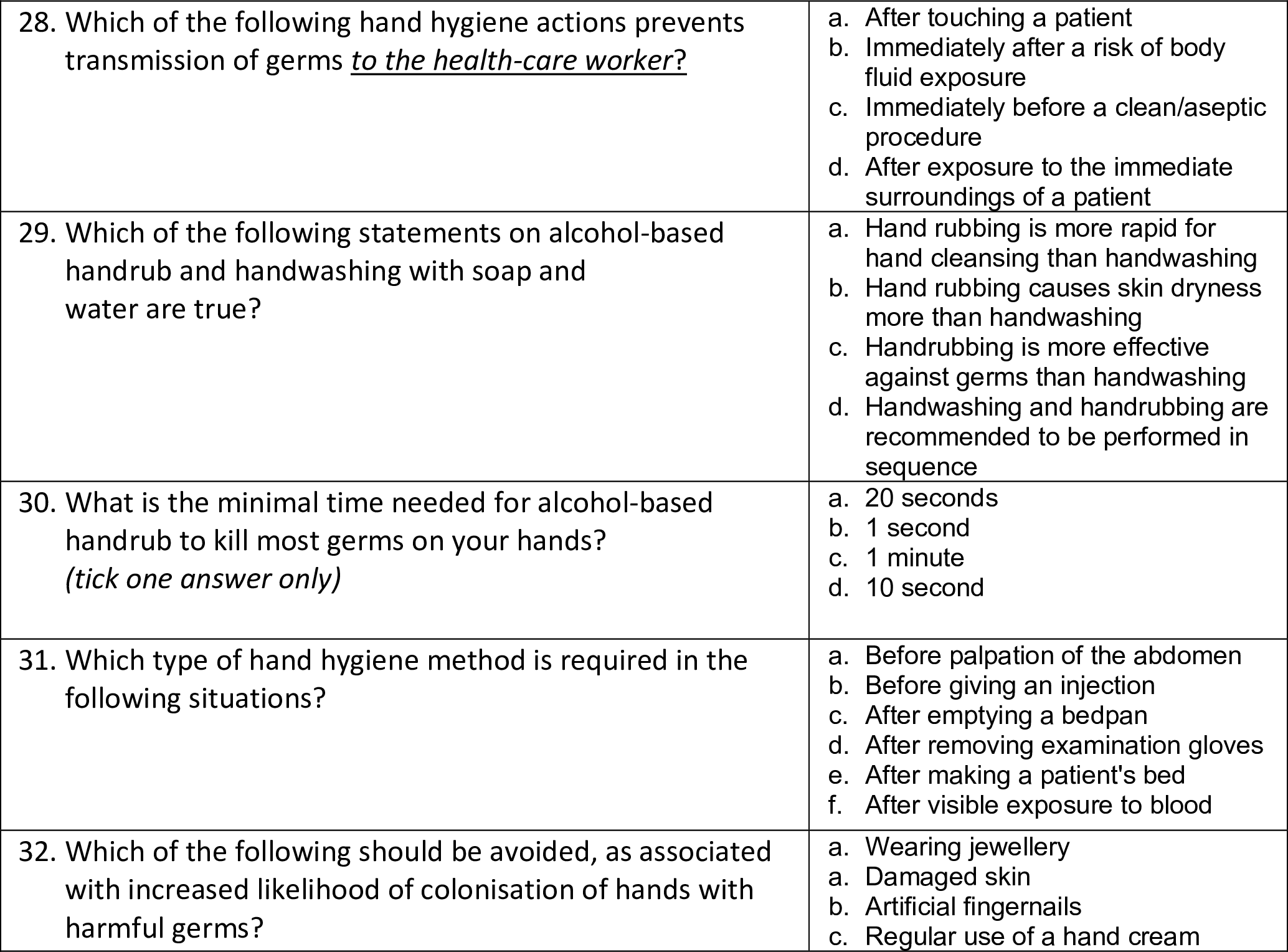

Supplement: S1 — (TIF) [file pone.0325323.s001.tif]
